# Relationship of Dietary Intake of Omega-3 and Omega-6 Fatty Acids with Risk of Prostate Cancer Development: A Meta-Analysis of Prospective Studies and Review of Literature

**DOI:** 10.1155/2012/826254

**Published:** 2012-10-18

**Authors:** Michael E. Chua, Maria Christina D. Sio, Mishell C. Sorongon, Jun S. Dy

**Affiliations:** ^1^Institute of Urology, St. Luke's Medical Center, 1102 Quezon City, Philippines; ^2^ENT Department, St. Luke's Medical Center, 1102 Quezon City, Philippines; ^3^Preventive and Community Medicine, St. Luke's College of Medicine, 1102 Quezon City, Philippines

## Abstract

*Objective*. To determine the relationship between dietary omega-3 fatty acids (*n*-3 PUFA) and omega-6 fatty acids (*n*-6 PUFA) with prostate cancer risk from meta-analysis of prospective studies. *Design*. The literature retrieved from electronic biomedical databases up to June 2011 was critically appraised. General variance-based method was used to pool the effect estimates at 95% confidence interval. Heterogeneity was assessed by Chi^2^ and quantified by *I* ^2^. *Results*. Eight cohort studies were included for meta-analysis. *n*-3 PUFA, *n*-6 PUFA, and their derivatives were not significantly associated with risk of prostate cancer in general. A significant negative association between high dietary intake of alpha-linolenic acid (ALA) and prostate cancer risk (pooled RR: 0.915; 95% CI: 0.849, 0.985; *P* = 0.019) was noted. Likewise, a slightly positive association was noted on dietary long-chain *n*-3 PUFA, composed of eicosapentaenoic acid (EPA) and docosahexaenoic acid (DHA) with prostate cancer risk (pooled RR: 1.135; 95% CI: 1.008, 1.278; *P* = 0.036); however, when two other cohort studies with data of EPA and DHA, both analyzed separately, were included into the pool, the association became not significant (RR: 1.034; 95% CI: 0.973, 1.096; *P* = 0.2780). *Conclusion*. Intake of *n*-3 PUFA and *n*-6 PUFA does not significantly affect risk of prostate cancer. High intake of ALA may reduce risk of prostate cancer, while intake of long-chain omega-3 fatty acids does not have a significant effect.

## 1. Introduction

### 1.1. Prostate Cancer

Currently, prostate cancer presents as a significant health problem. It is the most commonly diagnosed cancer in men and the second leading cause to cancer-related death in males [1]. Considerable amount of epidemiologic and experimental data suggested that diet or lifestyle interventions could potentially prevent diseases such as cancer [2]. Preliminary research studies have also shown that certain aspects of diet may influence the risk of developing prostate cancer but this remains to be ascertained [3]. Some of the compounds of interest were the *n*-3 and *n*-6 polyunsaturated fatty acids (PUFA). Since the introduction of a family of unsaturated fatty acids, *n*-3 in 1990s and *n*-6 fatty acids in early 2000s, which were popularly referred to as omega-3 fatty acids and omega-6 fatty acids, respectively, they have become a major interest for study in their relationship with prostate cancer development [4]. Systematic reviews featuring relationship of components of omega fatty acids with prostate cancer development have yielded inconsistent findings [5–8]. However, these reviews had heterogeneity across studies and publication bias that were the reasons for inconsistent findings and questionable validity. 

Investigators of this study sought to reexamine the data by including only prospective studies from recently published reports involving human research in determining the effects of dietary omega fatty acids with its components on prostate cancer incidence. To ensure valid and reliable evidence, the present study specifically has aimed to give a systematic review through quality assessment of all available literatures regarding the association of omega fatty acids and prostate cancer. The result of this study may be helpful in establishing evidence-based practices for the urologist such as giving advice about diet modifications for high risk patient for prostate cancer development.

## 2. Method

### 2.1. Identification of the Literature

The investigators, with the help of a board-certified librarian, used electronic databases to identify published medical literatures about omega-3 fatty acids, omega-6 fatty acids, and prostate cancer. Literature search was not restricted by language. Electronic databases utilized were the following: MEDLINE, Unbound MEDLINE, EMBASE, Science Direct, OVID, and Cochrane Library, including the Cochrane Database of Systematic Reviews and the Cochrane Central Register of Controlled Trials (up to June 2011). All of these databases were searched using Firefox, Opera browser, and Explorer Windows. The following MEDLINE Medical Subject Heading (MeSH) terms “Omega-3 Fatty Acids” OR “Omega-6 Fatty Acids” AND “Prostate Neoplasm” were used. For EMBASE, Science Direct, OVID, and Cochrane Library searches, search terms were “prostate,” “cancer,” “carcinoma,” “neoplasm,” “tumor,” “omega,” “fatty acids,” and “polyunsaturated.” Reference lists of studies that met the inclusion criteria and review articles or textbooks of related topics were searched for potentially relevant titles. An external peer reviewer was asked to identify additional relevant studies that may not be included in the draft. Inquiry from industry/nutrition experts was done to obtain any unpublished data. 

### 2.2. Inclusion and Exclusion Criteria of Studies for Meta-Analysis

Studies included in the meta-analysis were the following: study that described the effects of dietary consumption of omega-3 and/or omega-6 fatty acids (with or without their derivatives) on prostate cancer incidence; study with prospective cohort study design with human study population; study that described the effects of exposure to omega-3 and/or omega-6 with different levels of exposure. Animal study and *in vitro* experimental studies were excluded because these laboratory results may not correlate well with *in vivo* human physiologic outcomes. Case-control studies were excluded because these were susceptible to methodological biases, particularly recall bias. Review articles and letters to the editors were also excluded because only collection of information and opinions were discussed. 

### 2.3. Evaluation of the Literature

Two physician reviewers (a urology resident and a general practitioner) independently evaluated the citations and abstracts. The reviewers flagged an article's title that focused on omega-3 fatty acids, omega-6 fatty acids, and prostate cancer. Any article that either reviewer flagged was ordered, including articles that had titles and abstracts with undetermined relevance. Both physician reviewers independently reviewed each article obtained to determine which article can be included in the study using a standardized screening form. The reviewers resolved any disagreements. All stages of the review were performed independently by two reviewers knowledgeable in principles of critical appraisal. The reviewers resolved their differences while senior physician resolved any unsettled disagreements. 

### 2.4. Critical Appraisal of the Literature and Data Extraction 

For the articles included in the study, two reviewers independently evaluated the quality of the study design and execution of cohort studies. The investigators used available information from the published article to critically appraise the validity of the study design by evaluating the representative recruitment of the population, the baseline characteristics of sample, measurements and ascertainment of cases and exposures, description of withdrawals and dropouts, validity and reliability of the measurements (questionnaires) used, adjustment for confounders, completeness of followup, calculation of effect size estimate either odds ratio (OR) or relative risks (RR), size of confidence interval, Bradford Hills criteria, and applicability of the studies. Each study was scored according to the recommendation of the National Health Service, UK, on critical appraisal and evaluation of descriptive (Cohort) studies [18]. Each included study was, then, independently graded by the same reviewers using Newcastle-Ottawa Quality Assessment Scale (NOQAS) from Cochrane Collaboration for cohort studies [19]. If any discrepancy of the rating was found, reviewers would discuss the differences noted from the study until both reviewers would have a mutual agreement of the score.

### 2.5. Data Summary and Statistical Analysis

For this paper, the investigators had constructed a detailed table (Table 1), one reviewer tabulated data from each study which was counterchecked by another reviewer. The reported RRs or ORs were used to estimate the risk ratio of prostate cancer specific mortality or prostate cancer incidence and its subcategories among highest and lowest dietary intake of omega-3 fatty acids, omega-6 fatty acids, and their components. Adjusted RR or OR and corresponding confidence intervals (CI) were preferred when available in the publication. If a cohort study was published several times at different dates, the most recent and comprehensive data were included. If an included study reported no estimated effect measurement and raw data for the calculation of RR or OR estimates, authors of the study were emailed requesting for the said data. The investigators used the general variance-based method to analyze the cohort studies, because variance estimates were based on adjusted measures of effect and using 95% CI for the adjusted measure. CI was used because confounding variables are not ignored and superior in pooling observational data [20]. Each study's risk ratios were converted to natural logarithms to stabilize the variances. The variance of the risk ratio was estimated from the CI. Before estimating the summary of risk ratios, test for heterogeneity was done using Cochran's chi-square test (*Q*) to assess the consistency of associations. In cases of heterogeneity (*P* < 0.1), sensitivity analysis was conducted by repeating the meta-analysis but excluding one study (from the lowest quality score to the highest) at a time to assess the influence of each individual study on the summary estimate. To quantify the extent of heterogeneity in the pooled studies, between-study variances (*I* ^2^) were done. The *I* ^2^ statistic described the proportion of total variance in estimates of the RR due to heterogeneity between studies. Homogeneous study has an *I* ^2^ value of 0 and a fixed effect model of analysis was applied. After the trial of removing a study after the other was done, the least degree of heterogeneity was noted. Reasons for the observed heterogeneity were determined. If the sensitivity analysis showed significant heterogeneity (*P* ≤ 0.10), random effect model was used for analysis instead of the fixed effects model [21]. Forest plots of the relative risks wherein the point estimate for each effect estimate was sized according to the inverse of the variance for each study were determined. In this study, the investigators used Comprehensive Meta-Analysis software by Biostat for statistical analysis of pooled data. Publication bias was examined by using Egger and Begg analysis and visual inspection of funnel plots of standard error intercept of RRs or ORs of prostate cancer incidents and its subcategories with the highest and the lowest omega fatty acids quantile intake [22].

## 3. Results

### 3.1. Selected Studies

**Figure 1:**
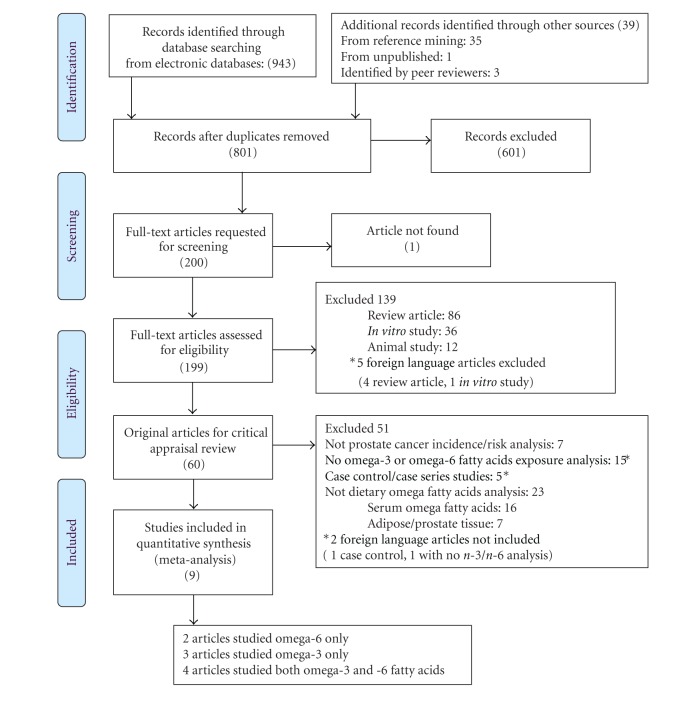
PRISMA diagram flow of literature search and study inclusion.

Results of the literature search are shown in Figure 1. Foreign language articles were included in the literature search; however, none met the inclusion criteria for the meta-analysis (corresponding author may be contacted for references of excluded studies). Eleven published articles were selected from a total of eight cohort studies [9–17, 23, 24]. This is because the result of one cohort, the Health Professionals Follow-Up Study, had four separate publications with different times of followup [11, 13, 23, 24]. Only the most recent or complete data source was included in the analysis. For ALA, data was extracted from Giovannucci et al. (2007) [13]. For other omega-3 FA derivatives, data were extracted from Leitzmann et al. (2004) [11]. Nine published articles were tabulated for their study characteristics [9–17] (Table 1) and included for statistical analyses. 

### 3.2. Study Characteristics

All studies included in the meta-analysis uniformly compared relative risk of prostate cancer incidence and prostate cancer specific mortality of involved population with the groups of highest intake and the lowest intake of FA. Fatty acid intake comparisons were either divided into quartile, tertile, or quintile groups. The age of the population ranges 40–55 years old at the initial phase of the study. Populations involved in the included studies were mainly from the western countries. All of the studies analyzed outcome using relative risk with incidence of prostate cancer. For ascertainment of cases, medical records, self-reports, cancer, and an endpoint biopsy was used. Follow-up period of the studies ranges 5–20 years. Omega fatty acid intake/exposure were determined by using food frequency questionnaire in all studies included. All of the studies adjusted their variables accordingly such as age, body mass index (BMI), smoking history, total energy intake, and family history of prostate cancer. 

### 3.3. Effect of Dietary Omega-3 and Omega-6 Intake on Risk of Prostate Cancer Development

 In general, total dietary intake of omega-3 and omega-6 has no significant relationship with prostate cancer development on total incidence; however, significant heterogeneity was noted on the analysis of ALA, EPA, DHA, and long-chain omega-3 fatty acids with total prostate cancer incidence (Table 2). In the sensitivity analysis for ALA, the Health Professionals Follow-up Study cohort by Giovannucci et al. (2007) weighted the major variation. Meta-analysis was repeated with the study replaced by an earlier published version (Leitzmann et al., 2004); heterogeneity was not significant (*P* = 0.227; *I* ^2^ = 29%) with pooled RR: 0.946; 95% CI: 0.885, 1.012; *P* = 0.105. It was also assessed in the quality analysis of studies that the Heath Professionals' Follow-Up Study cohort articles [11, 13] had the lowest scores; hence, repeat analysis was done by removing their data from the pooled effect estimates. Sensitivity analysis showed homogeneity among the remaining 4 cohort studies [9, 12, 15, 16] (*P* = 0.677;  *I* ^2^ = 0%) and the pooled effect estimates showed significant association (pooled RR: 0.915; 95% CI: 0.849, 0.985; *P* = 0.019) between high dietary ALA intake and prostate cancer risk (Table 3). The Begg (*P* = 0.30) and Egger (*P* = 0.34) tests, as well as visual inspection of the funnel plot, did not suggest a publication bias.

After excluding the cohort study of Health Professionals' follow-up in the sensitivity analysis for EPA and DHA, the remaining 3 cohort studies [9, 15, 16] with 151,326 men, showed no significant heterogeneity (*P* = 0.182; *I* ^2^ = 41%) and (*P* = 0.127;  *I* ^2^ = 51.5%); however, no significant association was found for high EPA intake (pooled RR for EPA: 1.049; 95% CI: 0.955, 1.152; *P* = 0.317) or high DHA intake (pooled RR for DHA: 1.032; 95% CI: 0.944; 1.128 *P* = 0.489) and prostate cancer risk (Table 3). The Begg (*P* = 0.60, *P* = 1.0), Eggers (*P* = 0.65, *P* = 0.54), and funnel plot did not show any publication bias. 

As aforementioned earlier, dietary long-chain omega-3 FA (combination of EPA and DHA) was used as a variable for subgroup analysis. If the Health Professionals' Follow-Up Study by Leitzmann et al. (2004) was removed due to its impact on heterogeneity, leaving 2 cohorts [16, 17] with 30,731 men involved, a significant positive association was noted between high dietary intake of long-chain omega-3 fatty acids and prostate cancer risk (pooled RR: 1.135; 95% CI: 1.008, 1.278; *P* = 0.036) (Table 3). Further analysis was done in this subgroup analysis by adding two cohorts [9, 15] that used EPA and DHA but did not combine these components as long chain omega-3 FA. Results of pooled effect estimates from 4 cohorts [9, 15–17] involving 82, 483 men showed no significant association between long-chain omega-3 FA and prostate cancer risk (pooled RR = 1.034; 95% CI: 0.973, 1.096; *P* = 0.2780) (Table 4). Despite of combining studies with differences in analysis, heterogeneity was not evident across the pooled data (*P* = 0.462;  *I* ^2^ = 0). Likewise, the Begg (*P* = 0.18; *P* = 0.26), Eggers (*P* = 0.26, *P* = 0.51) and funnel plot did not show any publication bias.

## 4. Discussion

The study explored available prospective studies regarding association of dietary omega-3 FA, omega-6 FA, and their components with prostate cancer risk. In general, the result of the meta-analysis does not show significant association between higher intakes of dietary omega-3 or omega-6 fatty acids, including their components with prostate cancer risk. Publication bias is also not significant. However, across the studies, heterogeneity is significant in most of the subgroup analysis (ALA, EPA, DHA, and long-chain omega-3 FA with total incidence of prostate cancer). The cohort studies of the Health Professionals Follow-Up Study [11, 13] contribute to the main weight of variability among the pooled studies. In the quality evaluation of the two reviewers, the said study had the lowest Newcastle-Ottawa quality score which may be due to biased selection of sample population which consisted only of health professionals. The same comment was raised by Carayol et al. (2010), who stated that health professionals compared to general population are more conscious of health conditions, while they also recognized that the exclusion of T1a cases in the cohort's analysis could be another contributing factor for bias [25]. In this meta-analysis, there were two other cohorts [12, 15] included in the pool, also excluded T1a cases in their study; hence, exclusion of T1a will not cause large interstudy variability. Despite the difference with that of the analysis by Carayol et al., we agreed that the cohort of Health Professionals Follow-Up Study is different from the rest of the included studies. It is not only due to the distinct population involved but also due to lower set levels of dietary ALA in their categorized highest quintile intake. Furthermore, in the initial three-year follow-up data from the said cohort [23] published in 1993, a significant positive association was already noted between ALA and prostate cancer risk. The same data were then carried over and have been included in the succeeding publication [13]. It was speculated that the assessment of dietary ALA may be inaccurate, because at that time in the United States during the late 80s to early 90s, known sources of ALA were unhealthy (e.g., canola oil in the form of mayonnaise and creamy salad dressings) and not from the later familiar sources of ALA (e.g., flaxseed, walnuts, and canola oil) [26]. This may be suggestive of a less healthy lifestyle relating to an increased risk of prostate cancer independent of ALA intake levels. Additionally, the multivariate adjustment of this cohort for BMI was significantly different from the rest of cohort studies. Participants included in the study were more than 40 years of age and tasked to recall their BMI when they were 21 years of age; which further suggests possibility of recall bias. Lastly, this cohort is the only study that used a long-term cumulative dietary model to assess omega FA intake by assessing dietary intake every four years with followup of 14 years [11] and 16 years [13], respectively. 

When repeat meta-analysis was done after removing the data of Health Professional Follow-Up Studies from the pool, sensitivity analysis improved significantly and homogeneity was noted among the studies (Cochran's chi-square test *P* > 0.1). With this modification, a significant negative association was noted between high dietary intake of ALA and total prostate cancer risk (pooled RR: 0.915; 95% CI: 0.849, 0.985; *P* = 0.019), which is in contrast with previous meta-analyses [27, 28]. Meta-analysis by Carayol et al. also suggested weak protective effect on prostate cancer risk by dietary intake of ALA; however, this was only detected when an alternate method of analysis was done. Their relative risk determination was set at a cut-off point value of 1.5 gm/day, and analysis was done by comparing groups consuming above versus below the cut-off point [25]. A study by Demark-Wahnefriend et al. also suggested that flaxseed-supplemented (30 g/day), containing high ALA, and fat restricted (20% of kilocalories) diet not only decreased serum cholesterol and derivatives but also resulted in a significant protective effect on tumor proliferation rate that was measured by biopsies done after 30 days of therapy [29]. Similar effect was noted by Freeman et al., which measured prostatic levels of fatty acids; which served as an estimate of fatty acid exposure of a target organ that likely reflects long-term dietary intake. They found that the ALA percentage was significantly lower when the tumor had extended to an anatomical or surgical margin [30]. 

A significant positive association was also noted between high long-chain omega-3 (EPA + DHA) fatty acids intake and total prostate cancer risk (pooled RR: 1.135; 95% CI: 1.008, 1.278; *P* = 0.036). This result was generated from two cohort studies [16, 17] that have a small population size relative to the other pooled studies. However, when two other cohort studies [9, 15] with different method of EPA and DHA analysis were added to the existing pool; the association was no longer significant (pooled RR = 1.034; 95% CI: 0.973, 1.096; *P* = 0.2780). As mentioned earlier, the small population size of the two cohort studies [16, 17], in the initial analysis, may account for the significant positive association initially seen. Both authors [16, 17] also mentioned that the positive association found may be due to a possible detection bias. The inclusion of the two other cohorts [9, 15] in the analysis resulted in an insignificant association which further supports the notion raised regarding small population size. 

In contrast to the previous meta-analyses, the investigators in this study believed that only prospective cohorts can produce better evidence to conclude any relationship between diet and cancer development. Moreover, this study used stricter methods in selecting and screening studies by applying the quality assessment scale recommended by the Cochrane Collaboration and the Newcastle-Ottawa Quality Assessment Scale (NOQAS) for cohort studies [19]. Aside from a wide range of subgroups which analyzed in this meta-analysis, adjustment was also done to maintain homogeneity of pooled effect estimates in instances where heterogeneity was found. The only limitation of this meta-analysis is the unavailability of randomized control trials (RCT), because none were found probably due to ethical reasons in doing trial in such methodology. During the hand searching process, only local studies were obtained because of some technical difficulties in obtaining internationally unpublished studies. Lastly, detection bias was not fully minimized by all included studies, since most of the cohorts did not perform endpoint biopsies of all involved subjects to completely rule out absence of prostate cancer.

## 5. Conclusion

 Dietary intake of omega-3 fatty acids and omega-6 fatty acids is not significantly associated with risk of prostate cancer, either local or advanced stage or low- or high-grade tumors, and prostate cancer specific mortality. Components of omega-6 fatty acids showed no significant association to prostate cancer risk. High dietary intake of alpha-linolenic acid may significantly reduce prostate cancer risk. Intake of long-chain omega-3 fatty acids does not have a significant effect. More good quality research is needed in determining effect of long-chain omega-3 fatty acids on prostate cancer incidence, grade, and specific mortality. 

## Figures and Tables

**Table 1:** Detailed summary of included prospective cohort studies and each study quality scoring.

								Quality scores			
								NOQAS			Critical appraisal —UK (11)
								(S)election			
Study author (year)	Population of study	Years of followup	Ascertain of cases (prostate cancer)	Omega fatty acid intake determination	Fatty acid omega-3/omega-6	Comparison of group used	Adjustment variables	(C)omparison			
								(O)utcome			
								S (4)	C (2)	O (3)	
Schuurman et al. [9], 1999	58, 279 men 55–69 yo	6.3 years	Pathology reports	150-item semiquantitative food frequency questionnaire (FFQ)	Omega 3 (ALA, EPA, DHA) and Omega 6 (LA, AA)	Quintile	Age, family history of prostate carcinoma, socioeconomic status, total energy intake	4	2	3	8

Laaksonen et al. [10], 2004	2,002 men 42–60 yo	12.6 years	Cancer registries	4-day food records nutrition estimated for fatty acids	Omega 6 (LA)	Tertile	Age, dietary intake of energy-adjusted saturated fat, fiber and calcium intake; plasma lipid-standardized tocopherol levels. BMI, fasting serum insulin and blood glucose levels and nonesterified fatty acid concentrations	4	2	3	9

Leitzmann et al. [11], 2004	44,856 men 40–75 yo	14 years	Self-report and medical/pathology record	Every 4 years 131-FFQ	Omega 3 and Omega 6 and ALA	Quintile	Age, major ancestry, family history of prostate cancer, BMI at age 21 y, height, type 2 diabetes, history of vasectomy, cigarette smoking, physical activity, intake of total energy, intakes of calcium, supplemental vitamin E, and lycopene.	3	2	3	8

Koralek et al. [12], 2006	29,592 men 55–74 yo	5.1 years (average years)	Self-report with medical record	Two 24 hr continuing Surveies of Food Intake by Individual over a year using 137 semiquantitative FFQ (ALA composite per food item)	Omega 3 (ALA)	Quintile	Age, current body mass index, family history of prostate cancer, history of diabetes, smoking history, intake of total energy, lycopene, supplemental vitamin E, aspirin use, physical activity, and race	4	2	3	8

Giovannucci et al. [13], 2007	51,529 40–75 yo	16 years	Self-report and hospital record	Every 4 years, 131 item semiquantitative FFQ	Omega 3 (ALA)	Quintile	Age, BMI at age 21 years, height, smoking, physical activity level, family history of prostate cancer, history of diabetes mellitus, race, and intakes of total calories, processed meat, fish, tomato sauce, and vitamin E supplements	3	2	3	8

Neuhouser et al. [14], 2007	18,314 smoker 50–62	10 years	Cancer registries	Every 2 years, self-assessment diet FFQ	Omega 6	Quartile	Age, race/ethnicity, energy intake, BMI, smoking and family history	3	2	3	8

Park et al. [15], 2007	82,483 men >45 yo	8 years	Cancer registries	Self-administered quantitative FFQ, 180 item 3-day record	Omega 3 (DHA and EPA) ALA Omega 6	Quintile	Age, ethnicity, family history of prostate cancer, education, BMI, smoking status, and energy intake	4	2	3	10

Wallstr$\ddot{\text{o}}$m et al. [16], 2007	10,564 men 45–73 yo	11 years	Cancer registry	Modified diet history seven-day menu book/168 item questionnaire, 45 min interview	Omega 3 and Omega 6	Quintile	Age, diabetes, waist circumference, height, educational level, alcohol habits, BMI, smoking history, birth country, total calcium intake, consumption of fruits, vegetables, and red meat All dietary variables were energy adjusted	4	2	3	10

Chavarro et al. [17], 2008	20,167 men 40–84 yo	19 years (mean year)	Medical record	12 months abbreviated food (fish) frequency questionnaire	Omega 3 (long-chain *n*-3)	Quintile	Age, BMI, physical activity, intakes of alcohol, tomato products, dairy products, and meat, smoking, race, use of multivitamins, use of vitamin E supplements	3	2	3	8

**Table 2:** Meta-analysis statistical summary on relationship of dietary omega 3 and omega 6 compound with prostate cancer risk.

Dietary compound intake	Number of studies (population size)	Total prostate cancer incidence	Sensitivity analysis
Total omega 3	2 studies (93,047)	RR: 0.973; 95% CI: 0.888, 1.065; *P* = 0.549	*P* = 0.264; *I* ^2^ = 20%
ALA	5 studies (228,668)	RR: 0.956; 95% CI: 0.855, 1.070; *P* = 0.436	*P* = 0.028; *I* ^2^ = 63%
ALA (adjusted)	4 studies (177,133)	RR: 0.915; 95% CI: 0.849, 0.985; *P* = 0.019	*P* = 0.677; *I* ^2^ = 0%
EPA	4 studies (196,192)	RR: 0.996; 95% CI: 0.921, 1.076; *P* = 0.911	*P* = 0.055; *I* ^2^ = 61%
EPA (adjusted)	3 studies (151,326)	RR: 1.049; 95% CI: 0.955, 1.152; *P* = 0.317	*P* = 0.182; *I* ^2^ = 41%
DHA	4 studies (196,192)	RR: 0.990; 95% CI: 0.918, 1.068; *P* = 0.804	*P* = 0.070; *I* ^2^ = 58%
DHA (adjusted)	3 studies (196,192)	RR: 1.032; 95% CI: 0.944, 1.128; *P* = 0.489	*P* = 0.127; *I* ^2^ = 52%
Long-chain *n*-3	3 studies (75,597)	RR: 1.058; 95% CI: 0.876, 1.280; *P* = 0.557	*P* = 0.023; *I* ^2^ = 73%
Long-chain *n*-3 (adjusted)	2 studies (30,731)	RR: 1.135; 95% CI: 1.008, 1.278; *P* = 0.036	*P* = 0.249; *I* ^2^ = 25%
Long-chain *n*-3 + (DHA + EPA)	4 studies (82,483)	RR: 1.034; 95% CI: 0.973, 1.096; *P* = 0.278	*P* = 0.462; *I* ^2^ = 0%
Total omega 6	3 studies (111,361)	RR: 1.038; 95% CI: 0.951, 1.133; *P* = 0.404	*P* = 0.576; *I* ^2^ = 0%
Linoleic acid	4 studies (115,711)	RR: 0.972; 95% CI: 0.859, 1.101; *P* = 0.659	*P* = 0.170; *I* ^2^ = 40%
Arachidonic acid	3 studies (113,709)	RR: 1.093; 95% CI: 0.973, 1.226; *P* = 0.134	*P* = 0.829; *I* ^2^ = 0%

**Table 3:** Forest plot of dietary alpha-linolenic acid (ALA), docosahexaenoic acid (DHA), eicosapentaenoic acid (EPA) intake and long-chain omega-3 intake versus total incidence of prostate cancer (Giovannucci et al. [11] removed).

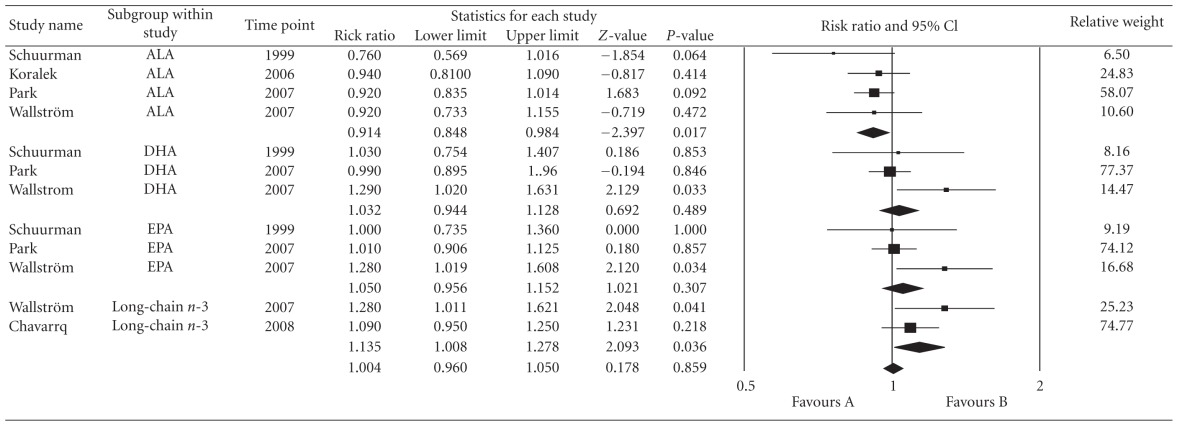

**Table 4:** Forest plot of dietary long-chain omega-3 fatty acid intake and total incidence of prostate cancer (Schuurman et al. [9]; Park et al. [15]; Wallstr$\ddot{\text{o}}$m et al. [16]; Chavarro et al. [17]).

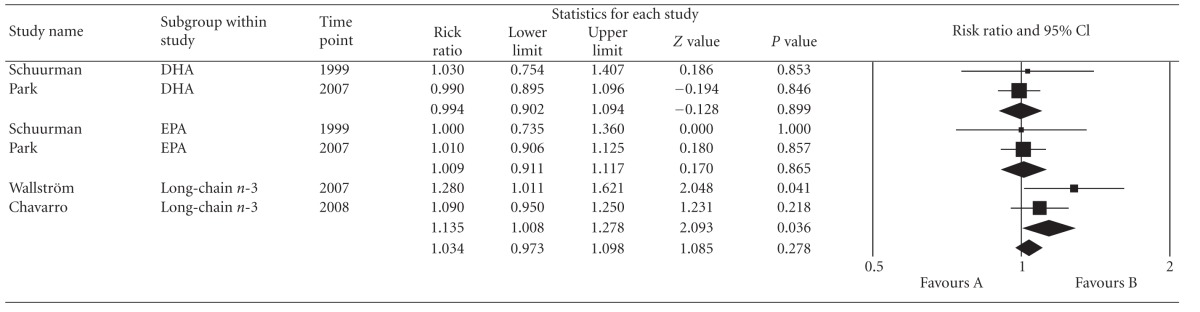
